# Monitoring the Sphingolipid *de novo* Synthesis by Stable-Isotope Labeling and Liquid Chromatography-Mass Spectrometry

**DOI:** 10.3389/fcell.2019.00210

**Published:** 2019-10-01

**Authors:** Dominik Wigger, Erich Gulbins, Burkhard Kleuser, Fabian Schumacher

**Affiliations:** ^1^Department of Toxicology, University of Potsdam, Nuthetal, Germany; ^2^Department of Molecular Biology, University of Duisburg-Essen, Essen, Germany; ^3^Department of Surgery, University of Cincinnati, Cincinnati, OH, United States

**Keywords:** sphingolipid *de novo* synthesis, serine palmitoyltransferase, mass spectrometry, stable-isotope labeling, ceramides

## Abstract

Sphingolipids are a class of lipids that share a sphingoid base backbone. They exert various effects in eukaryotes, ranging from structural roles in plasma membranes to cellular signaling. *De novo* sphingolipid synthesis takes place in the endoplasmic reticulum (ER), where the condensation of the activated C_16_ fatty acid palmitoyl-CoA and the amino acid L-serine is catalyzed by serine palmitoyltransferase (SPT). The product, 3-ketosphinganine, is then converted into more complex sphingolipids by additional ER-bound enzymes, resulting in the formation of ceramides. Since sphingolipid homeostasis is crucial to numerous cellular functions, improved assessment of sphingolipid metabolism will be key to better understanding several human diseases. To date, no assay exists capable of monitoring *de novo* synthesis sphingolipid in its entirety. Here, we have established a cell-free assay utilizing rat liver microsomes containing all the enzymes necessary for bottom-up synthesis of ceramides. Following lipid extraction, we were able to track the different intermediates of the sphingolipid metabolism pathway, namely 3-ketosphinganine, sphinganine, dihydroceramide, and ceramide. This was achieved by chromatographic separation of sphingolipid metabolites followed by detection of their accurate mass and characteristic fragmentations through high-resolution mass spectrometry and tandem-mass spectrometry. We were able to distinguish, unequivocally, between *de novo* synthesized sphingolipids and intrinsic species, inevitably present in the microsome preparations, through the addition of stable isotope-labeled palmitate-d_3_ and L-serine-d_3_. To the best of our knowledge, this is the first demonstration of a method monitoring the entirety of ER-associated sphingolipid biosynthesis. Proof-of-concept data was provided by modulating the levels of supplied cofactors (e.g., NADPH) or the addition of specific enzyme inhibitors (e.g., fumonisin B_1_). The presented microsomal assay may serve as a useful tool for monitoring alterations in sphingolipid *de novo* synthesis in cells or tissues. Additionally, our methodology may be used for metabolism studies of atypical substrates – naturally occurring or chemically tailored – as well as novel inhibitors of enzymes involved in sphingolipid *de novo* synthesis.

## Introduction

Sphingolipids are a lipid class of great physiological importance to the homeostasis of mammalian cells, eukaryotes as a whole, and some prokaryotes. Structurally, all sphingolipids share a C_18_ amino alcohol backbone, to which fatty acids of different chain lengths and head groups of varied polarity can be attached. Due to their amphipathic character, sphingolipids play significant roles in membrane biology, including the maintenance of barrier function and fluidity (Breslow and Weissman, 2010). Beyond this, sphingolipids include bioactive species that are involved in numerous cellular signaling cascades such as ceramides (Cer), sphingosine (Sph, synonymous with d18:1 Sph), and sphingosine 1-phosphate (S1P) (Hannun and Obeid, 2008). It has been shown that different bioactive sphingolipids can cause opposing biological effects. For instance, while Cer act pro-apoptotically (Obeid et al., 1993; Gulbins et al., 1995), S1P production correlates with proliferative (Olivera and Spiegel, 1993) and anti-apoptotic effects (Cuvillier et al., 1996). Concordantly, the relative levels of sphingolipid metabolites are key to cellular homeostasis and, as such, are tightly regulated, a process widely referred to as “sphingolipid rheostat” (Newton et al., 2015). Disturbances to sphingolipid metabolism are associated with numerous pathogenic states involved in, e.g., several cancers (Ogretmen, 2017), cardiovascular diseases (Borodzicz et al., 2015), metabolic diseases such as type 2 diabetes (Meikle and Summers, 2016), neuro-psychiatric disorders (Mühle et al., 2013), and bacterial and viral infections (Grassmé et al., 2003; Grassmé et al., 2005). To date, however, many mechanistic details regarding the involvement of sphingolipids in such pathologies remain poorly understood. Likewise, a complete understanding of the complex and highly interlinked metabolism of sphingolipids is still ongoing.

**FIGURE 1:**
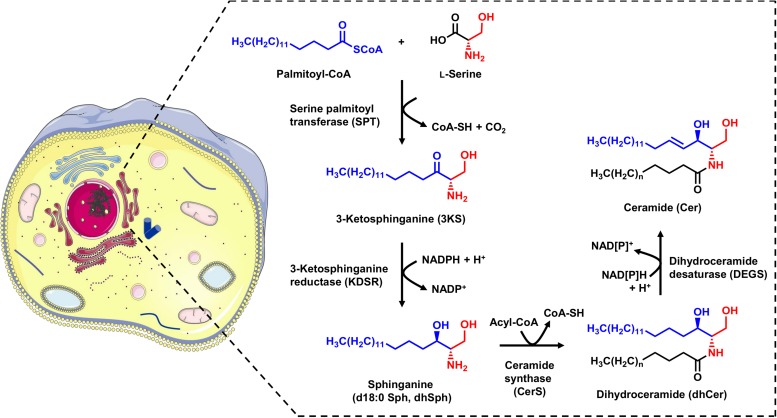
Schematic overview of the sphingolipid *de novo* synthesis located in the ER. Serine palmitoyltransferase (SPT) catalyzes the condensation of palmitoyl-CoA and L-serine. The product of this reaction, 3-ketosphinganine (3KS), is further NADPH-dependently reduced to sphinganine (dihydrosphingosine, d18:0 Sph) by 3-ketosphinganine reductase (KDSR). Ceramide synthases (CerS) couple fatty acyl-CoAs to the amino group of the long-chain base d18:0 Sph. This leads to the formation of dihydroceramides (dhCer), differing in the chain length of the amide-bound fatty acid. The final step is the introduction of a double bond between carbons C-4 and C-5 mediated by dihydroceramide desaturase (DEGS) under NAD[P]H consumption. The formed ceramides (Cer) are subsequently shuttled to the Golgi apparatus for further metabolism. The cell image (left) was taken from https://smart.servier.com (freely accessible).

Cer are the base molecules from which the synthesis of more complex sphingolipids, such as sphingomyelin and glycosphingolipids, takes place. They are also the precursors for bioactive lipid mediators such as Sph and S1P. Cer can be generated by sphingomyelin hydrolysis in membranes or *via* the catabolic salvage pathway, in which complex sphingolipids undergo lysosomal breakdown resulting in Cer (Hannun and Obeid, 2008). The *de novo* synthesis of Cer takes place in the endoplasmic reticulum (ER), and involves four enzymatic steps (Figure 1). The initial step is the condensation of the activated C_16_ fatty acid palmitoyl-CoA and the amino acid L-serine, which is catalyzed by pyridoxal 5’-phosphate (PLP)-dependent serine palmitoyltransferase (SPT). This produces 3-ketosphinganine (3KS), which is then rapidly reduced to sphinganine (dihydrosphingosine, d18:0 Sph) by 3-ketosphinganine reductase (also known as 3-ketodihydrosphingosine reductase, KDSR) in a NADPH dependent manner. Sphinganine, a so called long-chain base (LCB), is further *N*-acylated by the action of six ceramide synthase isoforms (CerS1-6) encoded by six distinct genes. Each CerS has a different acyl-CoA preference that can overlap within their isoforms. For example, CerS5 and CerS6 preferably use palmitoyl-CoA as their substrate, thus mainly forming C16 dihydroceramide (C16 dhCer, Cer d18:0/16:0) as a product. The last step in the sphingolipid *de novo* synthesis is catalyzed by dihydroceramide Δ4-desaturase (DEGS), which occurs in two isoforms: DEGS1 and DEGS2. Firstly, DEGS introduces a hydroxyl group at the C-4 position of the d18:0 Sph backbone using molecular oxygen. Next, a dehydration reaction follows in which DEGS, by means of NADPH (or NADH), introduces a double bond between carbons C-4 and C-5 to yield the Cer core-structure (Gault et al., 2010). For all enzymes involved in sphingolipid *de novo* synthesis (SPT, KDSR, CerS, and DEGS), there is strong evidence to suggest they are located on the cytosolic leaflet of the ER membrane (Gault et al., 2010; Yamaji and Hanada, 2015).

As with all enzymes involved in cellular metabolism, those regulating the sphingolipid biosynthesis can be affected by endogenous and exogenous factors as well as genetic alterations. Mammalian SPT is a heterodimer consisting of the two subunits SPTLC1 and SPTLC2 with the latter found as two isoforms (Tidhar and Futerman, 2013). Most patients suffering from hereditary sensory and autonomic neuropathy type 1 (HSAN1) carry mutations in *SPTLC1* or *SPTLC2*. These mutations do not affect the activity of the SPT-complex, but instead shift its substrate affinity so that in addition to canonical SPT products, cytotoxic 1-deoxysphingolipids are formed through the incorporation of L-alanine instead of L-serine. The disease phenotype varies with *SPT* mutations, producing a range of severe symptoms such as muscle atrophy, growth retardation, and lung complications (Bode et al., 2016). It was recently demonstrated that *KDSR* mutations causing structural and functional defects in KDSR, the next enzyme in sequence of *de novo* sphingolipid synthesis, have been shown to associate with the inherited skin disorder progressive symmetric erythrokeratoderma (Boyden et al., 2017). Likewise, genetic defects in *CerS* have been described and discussed as the cause of several human diseases. For instance, a missense coding single-nucleotide polymorphism in *CerS2* has been associated with rhegmatogenous retinal detachment (Kirin et al., 2013), while mutations in *CerS3* leading to impaired activity and expression of CerS3 have been linked to skin ichthyosis (Eckl et al., 2013). In addition, there are numerous examples in which changes in the expression or activity of CerS isoforms correlate with human diseases, cancers in particular. Subsequently, the use of CerS as therapeutic targets is currently under intense scrutiny (Park et al., 2014). Recently, a genetic defect in *DEGS1*, in conjunction with substantial increases of dihydroceramides in fibroblasts and muscles, was identified as causative of hypomyelinating leukodystrophy (Pant et al., 2019). Of therapeutic relevance, several modulators of DEGS1 activity have been identified, as summarized by Rodriguez-Cuenca et al. (2015). These include the DEGS1 inducers palmitate and interleukin-2, and the DEGS1 repressors vitamin E and oxidative stress.

All enzymes involved in the *de novo* synthesis of sphingolipids can be modulated and can act as central “crossroads” in numerous human diseases. Techniques that assess the affinities and activities of the individual enzymes are, therefore, of great importance. Since each step of ER-centered biosynthetic pathways influences the next, it is essential to empirically capture the complete sphingolipid *de novo* synthesis. As far as we know, there is currently no cell-free assay available capable of tracking the status of the SPT substrates palmitate and L-serine from the initial condensation product up to their incorporation within the Cer scaffold. Here, we present a microsomal *in vitro* assay using stable-isotope labeled starting materials (palmitate-d_3_ and L-serine-d_3_) along with state-of-the-art detection techniques [high-performance liquid chromatography (HPLC) coupled to high-resolution and tandem-mass spectrometry] for unambiguous monitoring sphingolipid biosynthetics within the ER in its entirety. Doing so, we were able to track the incorporation of palmitate and serine beyond the formation of LCBs to (dh)Cer.

## Materials and Methods

### Chemicals and Reagents

Palmitic acid (16,16,16-d_3_) was obtained from Cortecnet (Voisins-le-Bretonneux, France) and L-serine (2,3,3-d_3_) was from CDN Isotopes (Pointe-Claire, Canada). Cer d18:0/16:0 (C16 dhCer), Cer d18:1/16:0 (C16 Cer), Cer d18:1/17:0 (C17 Cer), d18:0 Sph (dhSph) and d17:0 Sph were from Avanti Polar Lipids (Alabaster, United States). 4-(2-hydroxyethyl)-1-piperazineethanesulfonic acid (HEPES), dimethyl sulfoxide (DMSO), disodium hydrogen phosphate dihydrate (Na_2_HPO_4_), dithiothreitol (DTT), fatty acid-free bovine serum albumin (BSA), glacial acetic acid, glycerol, magnesium chloride hexahydrate (MgCl_2_), potassium chloride (KCl), sodium chloride (NaCl), sodium dihydrogen phosphate monohydrate (NaH_2_PO_4_), sodium hydroxide (NaOH) and tris(hydroxymethyl)aminomethane (Tris) were purchased from Carl Roth (Karlsruhe, Germany). Potassium hydroxide was from Merck KGaA (Darmstadt, Germany). Coenzyme A (CoA), fumonisin B_1_ (FB_1_) and nicotinamide adenine dinucleotide phosphate sodium salt (NADPH) were received from Cayman Chemicals (Ann Arbor, United States). Adenosine 5′-triphosphate disodium salt hydrate (ATP), β-glycerophosphate disodium salt hydrate, calcium chloride dihydrate (CaCl_2_), cOmplete^TM^ Protease Inhibitor Cocktail, myriocin from *Mycelia sterilia*, nicotinamide adenine dinucleotide dipotassium salt (NADH), phenylmethylsulfonyl fluoride (PMSF), pyridoxal 5′-phosphate hydrate (PLP), *sn*-glycerol 3-phosphate lithium salt and sodium orthovanadate (Na_3_VO_4_) were ordered from Sigma-Aldrich (Taufkirchen, Germany). All solvents and additives used were of LC-MS grade. 1-Butanol was from Merck KGaA, formic acid from VWR (Darmstadt, Germany) and acetonitrile as well as methanol from Carl Roth. Water was purified by a Millipore apparatus (Millipore GmbH, Darmstadt, Germany).

### Preparation of Rat Liver Microsomes

Liver tissue of adult, male Wistar rats (2 g wet weight) was homogenized in 6 mL ice-cold homogenization buffer (10 mM NaH_2_PO_4_/Na_2_HPO_4_, 150 mM KCl, pH 7.4) using an Ultra Turrax T25 basic (IKA, Staufen, Germany). Cell debris were removed by centrifugation at 9,000 *g* for 20 min (4°C). To pellet microsomes, the supernatant was centrifuged for 1 h at 100,000 *g* (4°C) in a Beckman Coulter Optima LE-80K ultracentrifuge (Beckman Coulter, Krefeld, Germany) equipped with a Type 90 Ti fixed-angle rotor (Beckman Coulter). Microsomes were purified under the same conditions (1 h, 100,000 *g*, 4°C) in 4 mL homogenization buffer. After removal of the supernatant, the pellet was resuspended in microsomal buffer (5% (*w/v*) glycerol in homogenization buffer) and stored at −80°C. Protein concentration of the final microsome preparation was determined by Bradford assay.

### *In vitro* Sphingolipid *de novo* Synthesis Assay

The method was modified from a thin-layer chromatography-based protocol applied for investigations of *in vitro* S1P metabolism in membrane fractions published by Wakashima and co-workers (Wakashima et al., 2014). Rat liver microsomes (200 μg) were incubated with 0.3 mM palmitate-d_3_ and 4 mM L-serine-d_3_ for 1 h at 37°C under gentle shaking (120 rpm). The assay mixture with a total volume of 500 μL also contained 4 mM ATP, 5 mM β-glycerophosphate, 1 mM CaCl_2_, 160 μM CoA, cOmplete^TM^ Protease Inhibitor (2.7-fold), 0.8 mM DTT, 40 mM HEPES-NaOH, 2 mM MgCl_2_, 121 mM NaCl, 1 mM NADPH, 5 mM Na_3_VO_4_, 250 μM PLP, 0.8 mM PMSF and 1 mM *sn*-glycerol 3-phosphate (in the following referred to as “standard condition”). In order to modulate the sphingolipid *de novo* synthesis the following modifications of the standard protocol were applied: omission of NADPH, addition of 1 mM NADH, addition of 1 mM NADH and 5 μM FB_1_, addition of 1 mM NADH and 50 μM myriocin. Stock solutions of FB_1_, myriocin and PMSF were prepared in DMSO. All other substances were dissolved in water. To obtain an 8 mM palmitate-d_3_ stock solution, 41 mg were dissolved in 2 mL of 0.1 M NaOH at 75°C. Next, the solution was transferred at 55°C in 200 μL increments into 18 mL of a 10% BSA solution (in PBS). For the aqueous ATP solution, the pH had to be adjusted to 7.0 with Tris (1 M) to maintain stability at −20°C for 6 months. With the exception of CoA, NADPH and NADH that were freshly prepared on the day of the assay, stock solutions were stored at −20°C. Assay negative control samples were prepared under “standard conditions” with palmitate-d_3_ replaced by the vehicle BSA. *In vitro* sphingolipid biosynthesis was stopped by addition of 200 μL 1 M methanolic KOH. Sphingolipid extraction was carried out by addition of 1 mL 1-butanol (containing 20 pmol Cer d18:1/17:0 and 2 pmol d17:0 Sph as internal standards) and 0.5 mL of water-saturated 1-butanol. After extraction for 10 min under shaking (1,500 rpm) and centrifugation (2,300 *g*, 10 min, 4°C) the upper organic phase was neutralized with 16 μL glacial acetic acid and subsequently evaporated to dryness under reduced pressure using a Savant SpeedVac Concentrator (Thermo Fisher Scientific, Dreieich, Germany). Dried samples were dissolved in 100 μL of HPLC eluent mixture B/A (95:5, *v/v*, see next paragraph) (1,500 rpm, 10 min) and centrifuged at 2,300 *g* for 10 min (4°C). The supernatants were split into two vials and subjected to HPLC-MS analysis.

### Analysis of *de novo* Formed Labeled Sphingolipids by High-Performance Liquid Chromatography-Mass Spectrometry

All assay samples were subjected to HPLC coupled to both a quadrupole time-of-flight mass spectrometer (QTOF MS) and a triple quadrupole mass spectrometer (TQ MS). The rationale was to obtain accurate mass data (QTOF MS) as well as structural information by means of compound specific fragmentations with particularly high sensitivity (TQ MS). Chromatographic separations were performed with Agilent 1260 Infinity HPLC systems (Agilent Technologies, Waldbronn, Germany) coupled to the respective mass spectrometer, an Agilent 6530 QTOF MS or an Agilent 6490 TQ MS, *via* electrospray ion sources operating in the positive ion mode (ESI+). Samples (10 μL) were injected onto an Eclipse Plus C8 column (3.5 μm, 2.1 × 150 mm) guarded by a pre-column of the same material (both Agilent) that was tempered to 30°C. Water (eluent A) and methanol/acetonitrile (1:1, *v/v*, eluent B), both acidified with 0.1% formic acid, were used as eluents. Sphingolipid metabolites were eluted from the column by gradient elution using the settings given in Table 1. The total run time for one analysis was 30 min, including re-equilibration of the HPLC system. The following ion source parameters were used and, if not stated otherwise, maintained for both types of mass spectrometer used: drying gas temperature = 225°C, drying gas flow = 13 L/min of nitrogen (TQ MS: 15 L/min), sheath gas temperature = 380°C, sheath gas flow = 12 L/min of nitrogen, nebulizer pressure = 45 psi, capillary voltage = 4500 V, nozzle voltage = 2000 V. The ion funnel parameters (TQ MS only) were: high pressure RF voltage = 150 V and low pressure RF voltage = 60 V. The QTOF MS was operated in full scan mode, acquiring data in the mass-to-charge ratio (*m/z*) range of 100–750 with a scan rate of 2 spectra/s. TQ MS detection was accomplished in selected reaction monitoring (SRM) or, if applicable, in multiple reaction monitoring (MRM) mode. Fragmentations measured by collision-induced dissociation (CID) of investigated sphingolipids are given in Table 2. Most mass spectrometric results presented in this study are qualitative in nature. However, in experiments on modulation of sphingolipid biosynthesis *in vitro* (impact of enzyme inhibitors and cofactor supplementation), internal standards (d17:0 Sph and Cer d18:1/17:0) were used for semi-quantitative considerations. Furthermore, newly formed, labeled sphingolipids were quantified in technical assay replicates (using rat liver microsomes of the same batch) by means of d18:0 Sph, Cer d18:0/16:0, and Cer d18:1/16:0 as reference compounds for external calibration, as well as d17:0 Sph and Cer d18:1/17:0 as internal standards.

**TABLE 1:** HPLC gradient for separation of sphingolipids formed *de novo* in the microsomal assay^a^.

**Time (min)**	**Eluent A (%)^b^**	**Eluent B (%)^c^**	**Flow rate (mL/min)**
0	40	60	0.5
3	40	60	0.5
10	5	95	0.5
12	5	95	0.5
13	5	95	0.7
14	0	100	0.8
23	0	100	0.8
24	40	60	0.8
25	40	60	0.5

*^*a*^Analytical separation column: Eclipse Plus C8 (3.5 μm, 2.1 × 150 mm) + guard column of the same material (both Agilent Technologies). ^*b*^Water containing 0.1% formic acid. ^*c*^Methanol/acetonitrile (1:1, v:v) containing 0.1% formic acid.*

**TABLE 2:** MS/MS parameters for detection of *de novo* formed sphingolipids using the TQ MS system.

		**Collision**	**Dwell**
**Sphingolipid species**	**Mass transition (*m/z*)**	**energy (eV)**	**time (ms)**
3KS-d_5_	305.3 → 273.3	19	160
d18:0 Sph-d_5_	307.3 → 289.3	15	160
Cer d18:0-d_5_/16:0-d_3_	548.6 → 530.6	15	160
	548.6 → 289.3	25	160
Cer d18:1-d_5_/16:0-d_3_	528.6 → 269.3	25	160
**REFERENCE COMPOUNDS**			
d17:0 Sph (IS)^a^	288.5 → 270.5	12	160
Cer d18:1/17:0 (IS)^b^	534.5 → 264.3	25	160
d18:0 Sph	302.3 → 284.3	15	160
Cer d18:0/16:0	540.5 → 522.5	15	160
	540.5 → 284.3	25	160
Cer d18:1/16:0	520.5 → 264.3	25	160

*^*a*^Used as internal standard (IS) for labeled 3KS and d18:0 Sph. ^*b*^Used as IS for labeled C16 dhCer and C16 Cer.*

### Statistical Analysis

Differences in quantities of *de novo* formed sphingolipid intermediates generated *via* two different assay conditions were statistically evaluated by multiple *t*-tests (^∗∗∗^*p* < 0.001; n.s., non-significant) using the software GraphPad Prism Version 6.5 (GraphPad Software, Inc., La Jolla, United States).

## Results

### Formation of 3KS-d_5_ by the SPT-Catalyzed Reaction of d_3_-Palmitoyl-CoA and Serine-d_3_

**FIGURE 2:**
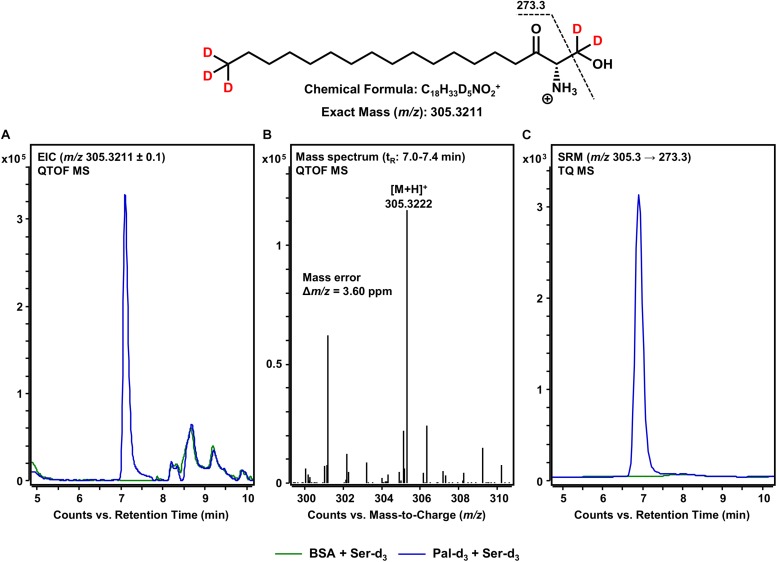
SPT-catalyzed formation of 3KS-d_5_ from the *in vitro* assay substrates palmitate-d_3_ and L-serine-d_3_. **(Top)** Chemical structure, formula, and exact mass of the protonated molecular ion [M+H]^+^ of 3KS-d_5_ generated during electrospray ionization. Dashed black line represents the mass transition used for selected reaction monitoring (SRM). **(A)** Overlay of extracted ion chromatograms (EIC) of the microsomal *de novo* synthesis assay using palmitate-d_3_ and serine-d_3_ as substrates (blue) compared to the negative control omitting palmitate-d_3_ (green). **(B)** Mass spectrum extracted from the EIC in the retention time window of eluting 3KS-d_5_ with annotation of the molecular ion peak and the calculated mass error. **(A,B)** Analysis was conducted on a quadrupole time-of-flight mass spectrometer (QTOF MS). **(C)** Overlay of SRM analyses of assay (blue) compared to negative control sample (green) performed with a triple quadrupole mass spectrometer (TQ MS). t_R_, retention time. **(A)** In the t_R_ range of 8–10 min, relatively abundant unidentified matrix components (*m/z*: 305.2211 ≤ × ≤ 305.4211) eluted from both the assay and negative control samples. **(C)** Concordant matrix signals were diminished, with increased specificity achieved by analyzing compound-specific fragmentation.

**FIGURE 3:**
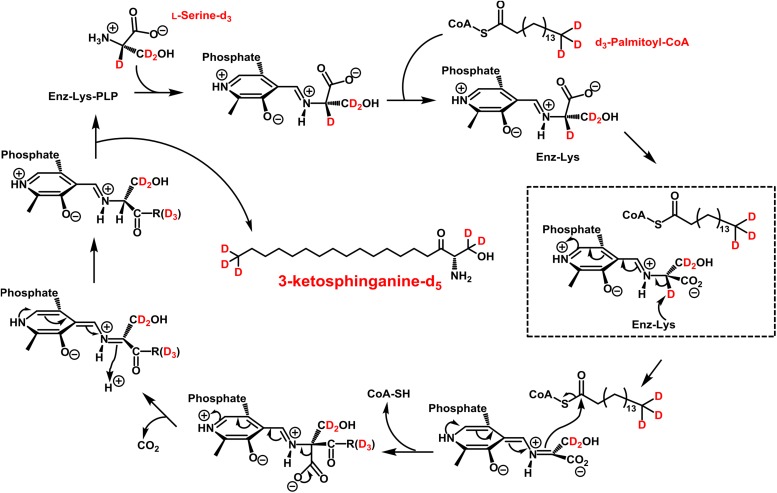
Proposed catalytic cycle for serine palmitoyltransferase (SPT) with d_3_-palmitoyl-CoA and L-serine-d_3_ being the substrates. This scheme was modified from A. H. Merrill, Jr. (Merrill, 2011). An internal aldimine (Schiff base) formed between the cofactor pyridoxal 5’-phosphate (PLP) and an active site lysine residue (Enz-Lys-PLP, upper left) is replaced by the external aldimine through the incoming L-serine-d_3_. Binding of d_3_-palmitoyl-CoA results in transfer of serine α-deuterium to an enzymatic lysine residue (dashed box). The reaction proceeds as shown with free CoA, CO_2_, and 3-ketosphinganine-d_5_ (3KS-d_5_) are released as products.

The first step in sphingolipid *de novo* synthesis is the SPT-catalyzed reaction of palmitoyl-CoA with L-serine, and the subsequent formation of 3KS (Figure 1). For the *in vitro* assay presented herein, palmitate-d_3_ and L-serine-d_3_ were applied as SPT substrates. A prerequisite for this strategy was the *in situ* activation of the fatty acid palmitate-d_3_. This was achieved by addition of CoA and ATP to the microsome preparations containing the fatty acyl-CoA synthetases (Normann et al., 1981) responsible for the formation of d_3_-palmitoyl-CoA. First, we ran the *in vitro* assay without the addition of NADPH in order to stop sphingolipid biosynthesis after the initial SPT catalysis. Thus, we were able to maximize product concentrations, as reducing equivalents are necessary for the subsequent step in sphingolipid biosynthesis. We analyzed the corresponding lipid extract by HPLC-QTOF MS (Figures 2A,B) and HPLC-TQ MS (Figure 2C). Using high-resolution MS, we observed a compound eluting at 7.1 min with the accurate *m/z* of 305.3222 that was not present in the negative control (complete assay with palmitate-d_3_ substituted by the vehicle BSA). This *m/z* matched the protonated molecular ion [M+H]^+^ of 3KS-d_5_ (chemical structure given in the topmost panel of Figure 2) with a mass error of only 3.60 ppm. At first glance, it seems surprising that the reaction of substrates, each of them labeled three-times, yields a product that is only quintuply labeled. However, a detailed consideration of the intensely studied SPT catalytic cycle reveals the fate of one particular deuterium atom, the α-deuterium located at C-2 position of the L-serine (2,3,3-d_3_) used as substrate. As illustrated in Figure 3, an internal aldimine (Enz-Lys-PLP) formed between the cofactor PLP and a lysine residue of the enzyme’s active site is replaced by the external aldimine composed of PLP and the incoming serine-d_3._ Binding of palmitoyl-CoA – in this case (16,16,16-d_3_)-palmitoyl-CoA – results in transfer of the α-deuterium from the external aldimine to an enzymatic lysine residue. This previously described deuterium loss (Ren et al., 2018) leads, after completion of SPT catalysis, to the formation of a five-times labeled 3KS, deuterated twice at the amino acid moiety and three times at the terminus of the fatty acid. To verify the formation of 3KS-d_5_ from palmitate-d_3_ and serine-d_3_ under the chosen experimental conditions, we subjected the lipid extract to HPLC-TQ MS in order to confirm structural identity by MS/MS. Cleavage of the terminal CH_2_O group has been reported as the predominant fragmentation of the natural SPT product 3KS, as reflected by the mass transition *m/z* 300.3 → 270.3 used for SRM analysis (Merrill et al., 2005). We transferred this structural information to the initial SPT product that is generated in our assay and, therefore, analyzed the CID of the terminal CD_2_O group of 3KS-d_5_. A clear signal for the SRM transition *m/z* 305.3 → 273.3, absent in the negative control (Figure 2C), further confirmed the proper function of the SPT and, thus, the first step of the presented *in vitro* sphingolipid *de novo* synthesis assay.

### *De novo* Formation of d18:0 Sph-d_5_ From 3KS-d_5_

**FIGURE 4:**
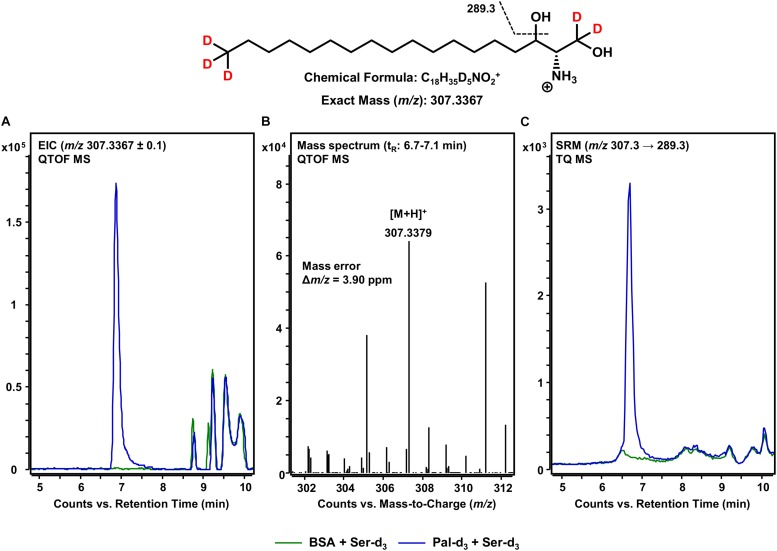
*De novo* formation of d18:0 Sph-d_5_ from 3KS-d_5_ in the *in vitro* assay. **(Top)** Chemical structure, formula, and exact mass of the protonated molecular ion [M+H]^+^ of d18:0 Sph-d_5_ generated during electrospray ionization. Dashed black line represents the mass transition used for selected reaction monitoring (SRM). **(A)** Overlay of extracted ion chromatograms (EIC) of the microsomal *de novo* synthesis assay using palmitate-d_3_ and serine-d_3_ as substrates (blue) compared to the negative control omitting palmitate-d_3_ (green). **(B)** Mass spectrum extracted from the EIC in the retention time window of eluting d18:0 Sph-d_5_ with annotation of the molecular ion peak and the calculated mass error. **(A,B)** Analysis was conducted on a quadrupole time-of-flight mass spectrometer (QTOF MS). **(C)** Overlay of SRM analyses of assay (blue) compared to negative control sample (green) performed with a triple quadrupole mass spectrometer (TQ MS). t_R_, retention time. **(A)** In the t_R_ range of 8.5–10 min, relatively abundant unidentified matrix components (*m/z*: 307.2367 ≤ × ≤ 307.4367) eluted from both the assay and negative control samples. **(C)** Concordant matrix signals were diminished, with increased specificity achieved by analyzing compound-specific fragmentation.

Following the formation of 3KS, sphingolipid biosynthesis proceeds with the reduction of 3KS to d18:0 Sph catalyzed by KDSR (Figure 1). Within this, the presence of NADPH is essential to the conversion of the C-3 keto group to a hydroxyl group. We thus performed our assay under “standard conditions” – in the presence of all cofactors necessary for full ER-based sphingolipid biosynthesis (see section Materials and Methods). Again, we performed accurate mass HPLC-QTOF MS with the extracted assay sample. Knowing that the presumable product should be five-times deuterated (chemical structure given in Figure 4), we extracted the [M+H]^+^ ion with *m/z* 307.3367 from the QTOF MS full scan. As can be seen in Figure 4A, an intense signal not present when palmitate-d_3_ was excluded from the assay reaction mixture, was detected. The corresponding mass spectrum (Figure 4B) confirmed the presence of d18:0 Sph-d_5_ with high mass accuracy (mass error less than 4 ppm). The slightly shorter retention time (6.9 min) compared to those observed for 3KS-d_5_ (7.1 min, Figure 2) is in agreement with the higher polarity of the hydroxyl compared to the keto group. Additionally, we performed MS/MS fragmentation of *de novo* synthesized d18:0 Sph-d_5_ using the TQ MS system. We monitored the mass transition *m/z* 307.3 → 289.3, as water loss accompanied by a mass shift of 18 Da has been reported during CID of unlabeled sphinganine (Cho et al., 2019). Indeed, a sharp SRM signal not seen in the negative control was obtained (Figure 4C). The data presented so far demonstrate the functionality of our novel microsomal assay over the first two steps of sphingolipid *de novo* synthesis starting from palmitate-d_3_ and serine-d_3_.

### Formation of Cer d18:0-d_5_/16:0-d_3_ From *de novo* Formed d18:0 Sph-d_5_ and Assay Component Palmitate-d_3_

**FIGURE 5:**
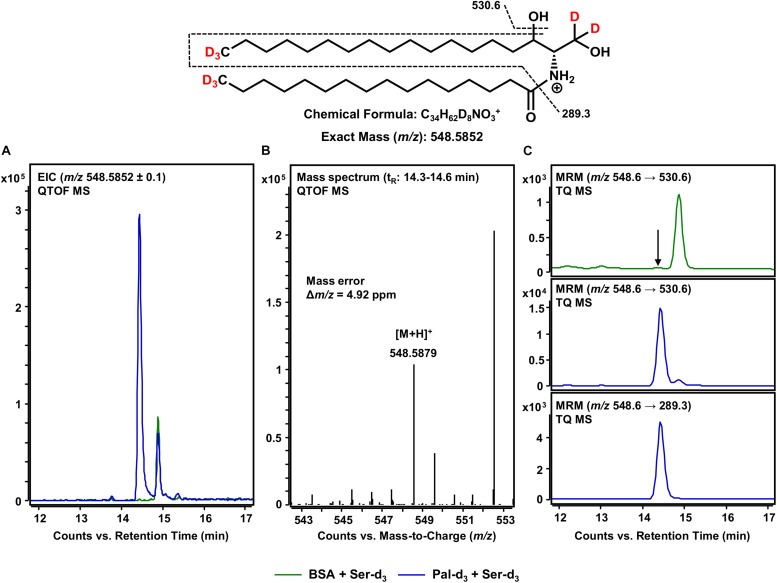
Formation of Cer d18:0-d_5_/16:0-d_3_ from *de novo* formed d18:0 Sph-d_5_ and assay substrate palmitate-d_3_. **(Top)** Chemical structure, formula, and exact mass of the protonated molecular ion [M+H]^+^ of Cer d18:0-d_5_/16:0-d_3_ generated during electrospray ionization. Dashed black lines represent the mass transitions used for multiple reaction monitoring (MRM). **(A)** Overlay of extracted ion chromatograms (EIC) from the microsomal *de novo* synthesis assay using palmitate-d_3_ and serine-d_3_ as substrates (blue), compared to the negative control omitting palmitate-d_3_ (green). **(B)** Mass spectrum extracted from the EIC in the retention time window of eluting Cer d18:0-d_5_/16:0-d_3_ with annotation of the molecular ion peak and the calculated mass error. **(A,B)** Analysis was conducted on a quadrupole time-of-flight mass spectrometer (QTOF MS). **(C)** MRM analyses of assay (blue) compared to negative control sample (green) performed with a triple quadrupole mass spectrometer (TQ MS). For the negative control, only the mass transition with highest intensity (*m/z* 548.6 → 530.6) is shown. The arrow indicates the retention time (t_R_) of Cer d18:0-d_5_/16:0-d_3_. **(A)** At t_R_ = 15 min, a relatively abundant unidentified compound (*m/z*: 548.4852 ≤ x ≤ 548.6852) eluted that was present in both assay and negative control samples. **(C)** Analysis of two analyte-specific fragmentations allowed for unambiguous discrimination between Cer d18:0-d_5_/16:0-d_3_ and matrix components.

After the formation of the sphingoid base backbone, biosynthesis of sphingolipids continues with the incorporation of a further fatty acid side chain (Figure 1). This central step is catalyzed by six different CerS that differ in their ability to incorporate fatty acids of varying chain lengths (Levy and Futerman, 2010). Within the here presented assay, both exogenous palmitate-d_3_ and those fatty acids inevitably contained within the microsomal preparation are available. We focused on the use of palmitate-d_3_ as a CerS substrate and, thus, on the production of Cer d18:0-d_5_/16:0-d_3_ as assessed by mass spectrometry. It should be noted here that the incorporation of any other fatty acid could be tracked in this same fashion. We analyzed samples of our *in vitro* assay for [M+H]^+^ ions with *m/z* 548.5852 corresponding to eightfold deuterated Cer d18:0-d_5_/16:0-d_3_ (also referred to as C16 dhCer-d_8_ for simplicity; chemical structure given in Figure 5). HPLC-QTOF MS analysis gave two adjacent signals in the retention time range between 14 and 15 min, with only one of them (14.4 min) not appearing in the negative control (Figure 5A). The mass spectrum of this particular signal revealed a molecular ion peak at *m/z* 548.5879 (Figure 5B) that corresponded to the sought product, with a mass error of not more than 4.9 ppm. To ensure that the correct biosynthetic product was detected, we performed an MRM analysis with the HPLC-TQ MS. At least two characteristic MS/MS fragmentations have been described for dihydroceramides. The first, loss of water at, presumably, the C-3 position of the sphingoid base backbone (Mi et al., 2016). The second, dehydration in conjunction with cleavage of the fatty acid bound to the amino group (Schiffmann et al., 2009). Both mass transitions – *m/z* 548.6 → 530.6 and *m/z* 548.6 → 289.3, respectively – were recorded in order to verify the presence of C16 dhCer-d_8_. Unlike the negative control (omission of palmitate-d_3_), clear signals were observed for both fragmentations (Figure 5C), co-eluting as expected and from which the loss of water gave the higher intensity. The successful detection of C16 dhCer-d_8_ represents the first documented *in vitro* cell-free tracking of sphingolipid biosynthesis starting from SPT substrates to the construction of the Cer scaffold. However, one further step is required to complete the *de novo* synthesis of sphingolipids.

### Detection of Cer d18:1-d_5_/16:0-d_3_ as the Terminal Product of the *in vitro* Sphingolipid *de novo* Synthesis Assay

**FIGURE 6:**
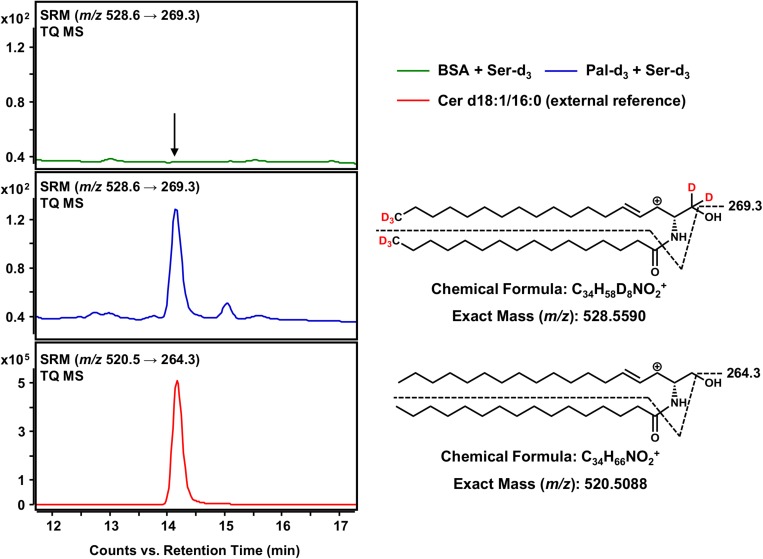
Detection of Cer d18:1-d_5_/16:0-d_3_ as the terminal product of the sphingolipid *de novo* synthesis assay. **(Right)** Chemical structures, formulas and exact masses of the dehydrated, protonated molecular ions [M-H_2_O+H]^+^ of Cer d18:1-d_5_/16:0-d_3_ and the unlabeled analog Cer d18:1/16:0, generated during electrospray ionization. Dashed black lines represent the mass transitions used for selected reaction monitoring (SRM). **(Left)** SRM analyses of assay sample (addition of palmitate-d_3_ and serine-d_3_, blue), negative control sample (omission of palmitate-d_3_, green) and external Cer d18:1/16:0 standard (red) were performed with a triple quadrupole mass spectrometer (TQ MS). The arrow indicates the retention time of Cer d18:1/16:0 (labeled and unlabeled).

The final step in the *de novo* synthesis of sphingolipids in the ER is the desaturation of dihydroceramide, leading to the formation of Cer (Figure 1). Here, DEGS introduces a double bond in the sphingoid base backbone between the C-4 and C-5 carbons, crucial to the biological activity of Cer (Bielawska et al., 1993; Brockman et al., 2004). As reported by us (Kachler et al., 2017) and others (Liebisch et al., 1999), Cer yield abundant [M-H_2_O+H]^+^ precursor ions during the ESI process. Hence, we conducted HPLC-QTOF MS for *m/z* 528.5590 ions representing Cer d18:1-d_5_/16:0-d_3_ (also referred to as C16 Cer-d_8_ for simplicity). As we were unable to obtain a signal clearly distinguishable from that of the negative control using HPLC-QTOF MS (not shown), we applied the technique of highest detection sensitivity to the assay samples. Using the HPLC-TQ MS system we analyzed the extracted assay sample for the major MS/MS fragmentation reported for Cer; the loss of the amide-bound fatty acid plus the dehydration of carbon C-1 of the sphingoid base (Liebisch et al., 1999), expressed as *m/z* 528.6 → 269.3 for C16 Cer-d_8_. Using this analytical approach, we could obtain an unambiguous signal for C16 Cer-d_8_, eluting at 14.1 min from the separation column (Figure 6), which was absent in the negative control. As one would expect, the introduction of a double bond shortened the retention time in the reversed-phase separation column as compared to the saturated C16 dhCer-d_8_ (Figure 5). To confirm our observation, we analyzed an unlabeled Cer d18:1/16:0 reference standard under identical instrumental conditions. This compound showed the same retention time as the C16 Cer-d_8_ formed *de novo*, as well as an analogous MS/MS fragmentation (*m/z* 520.5 → 264.3) (Figure 6). Although the obtained signal intensity for C16 Cer-d_8_ was comparatively small (the following section details a quantitative consideration in this regard), the MS-based tracking of labeled palmitate and serine through to the synthesis of Cer is, nevertheless, remarkable and has not previously been described.

### Regulation of Sphingolipid *de novo* Synthesis by Cofactor Supplementation and Addition of Enzyme Inhibitors

The biosynthesis of sphingolipids in the ER requires the activity of four different enzymes should the six CerS isoforms are considered as one, which themselves require the presence of several cofactors (Figure 1). By modification of the latter, it is possible to influence the progression of *de novo* sphingolipid synthesis at several points, a procedure we decided to perform to acquire proof-of-concept data regarding the here presented assay’s potential application. *In vitro* sphingolipid *de novo* synthesis was investigated under the conditions given in the Materials and Methods section with the following modifications: (1) omission of NADPH, (2) addition of NADPH (“standard condition”), (3) addition of NADPH and NADH, (4) as 3. with FB_1_, (5) as 3. with myriocin. With the described HPLC-MS methodology, we had a valuable tool to evaluate the individual assay conditions. It was initially decided to compare different assay conditions semi-quantitatively, using d17:0 Sph and Cer d18:1/17:0 as internal standards, in order to correct for analyte losses during sample preparation and for fluctuations of the mass spectrometer’s performance.

**FIGURE 7:**
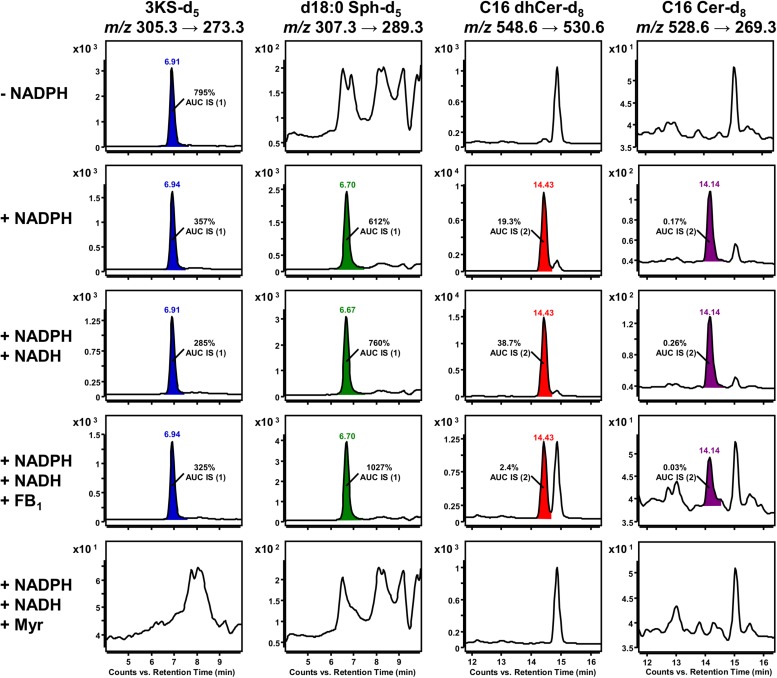
Impact of cofactor supplementation and presence of enzyme inhibitors on *de novo* formation of deuterated sphingolipid metabolites in the microsomal assay. Selected reaction monitoring (SRM) using a triple quadrupole mass spectrometer (TQ MS) was conducted to investigate the formation of 3KS-d_5_ (first column), d18:0 Sph-d_5_ (second column), Cer d18:0-d_5_/16:0-d_3_ (C16 dhCer-d_8_, third column) and Cer d18:1-d_5_/16:0-d_3_ (C16 Cer-d_8_, fourth column) under different assay conditions. The *in vitro* assay reaction mixture was prepared as described in section Materials and Methods with the following modifications: omission of NADPH (first row), “standard conditions” (with NADPH, second row), supplemented with NADPH and NADH (third to fifth row), addition of ceramide synthase (CerS) inhibitor fumonisin B_1_ (FB_1_, fourth row), and addition of serine palmitoyltransferase (SPT) inhibitor myriocin (Myr, fifth row). Signals that were not present in the corresponding negative controls were integrated and colored. d17:0 Sph (0.2 pmol injected on column) and Cer d18:1/17:0 (2 pmol injected on column), referred to as internal standards (IS) 1 and 2, respectively, were used to normalize areas under the curves (AUC). Corresponding relations are indicated as insets. Y axes are scaled differently, while x axes are maintained for each sphingolipid metabolite.

Standard conditions without NADPH are sufficient for the initial SPT-catalyzed reaction, as only CoA, ATP (for activation of palmitate-d_3_) and PLP (SPT cofactor) are required. Accordingly, a clear SRM signal for 3KS-d_5_ was detected. However, signals for the three downstream products of the *de novo* synthesis were missing (first row of Figure 7). NADPH is essential for the reduction of 3KS-d_5_ to d18:0 Sph-d_5_. Subsequently, *de novo* synthesis stops after the SPT reaction when NADPH is absent. By contrast, NADPH or NADPH/NADH supplementation enables the sphingolipid biosynthesis to reach its final stage, the formation of C16 Cer-d_8_ (second and third row of Figure 7). In addition to the formation of d18:0 Sph, NADPH is also required for the desaturation of dhCer (Figure 1). For the latter step, NADH has also been described as a reduction agent (Michel et al., 1997), which is the rational for the integration of this component into our assay. CerS, enzymes central to sphingolipid *de novo* synthesis, can be competitively inhibited by FB_1_, a mycotoxin structurally related to sphingosine (Wang et al., 1991). The presence of FB_1_ drastically reduced the abundance of C16 dhCer-d_8_ and subsequently C16 Cer-d_8_ (Figure 7, fourth row). The addition of myriocin completely prevented the formation of deuterated sphingolipids (Figure 7, fifth row). This natural fungal product is a potent SPT inhibitor (Miyake et al., 1995) and thus suppresses the condensation of d_3_-palmitoyl-CoA and L-serine-d_3_.

Peak areas of detected *de novo* formed, labeled sphingolipids were related to those of internal standards (d17:0 Sph for 3KS-d_5_, d18:0 Sph-d_5_ and Cer d18:1/17:0 for C16 dhCer-d_8_, C16 Cer-d_8_) added prior to lipid extraction. Internal standard areas under the curves (AUCs) were stable throughout the assays, with standard deviations of less than 10%. The presence of both NADPH and NADH in the assay mixture increased signals of d18:0 Sph-d_5_ (1.2-fold), C16 dhCer-d_8_ (2.0-fold), and C16 Cer-d_8_ (1.5-fold) compared to solely NADPH. Concordantly, the 3KS-d_5_ signal also decreased (by 20%). Inhibition of CerS by FB_1_ increased the amount of substrate d18:0 Sph-d_5_ to 135% and drastically diminished the product C16 dhCer-d_8_ by more than 90%. As expected, the formation of the consecutive product, C16 Cer-d_8_, was reduced similarly in the presence of FB_1_. The 3KS-d_5_ signal was highest in samples without reducing equivalents, where *de novo* synthesis was stopped after SPT catalysis. In all other samples (except in presence of myriocin) intensity of d18:0 Sph-d_5_ exceeded those of 3KS-d_5_ (1.7–3.2-fold). Unexpectedly, C16 dhCer-d_8_ was detected in significantly greater quantities than C16 Cer-d_8_. On a semi-quantitative basis, this difference ranged between 80 and 149-fold. In assay samples containing myriocin, signals of the internal standards were detected, while those of *de novo* formed deuterated sphingolipids were not.

**FIGURE 8:**
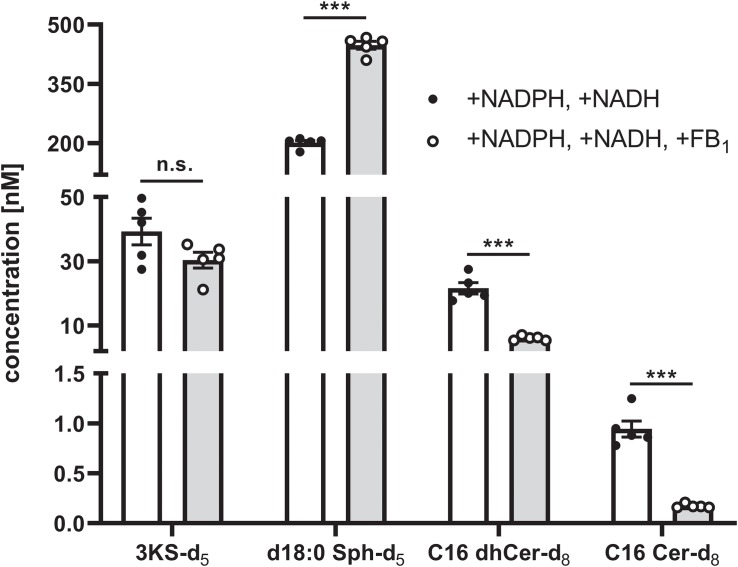
Intra-assay variability of the sphingolipid *de novo* synthesis assay. A total of 10 technical assay replicates were prepared, five of which were supplemented with NADPH and NADH (white bars and solid circles), while the remaining five replicates were additionally with fumonisin B_1_ (FB_1_, gray bars and open circles). Assays were performed as described in the Materials and Methods section using identical batches of microsomes and reagents. Concentrations of *de novo* formed, deuterated sphingolipid metabolites in the final sample volume (100 μL) were quantified by HPLC-MS/MS using external calibration (concentration range: 0–1000 nM). To this end, 3KS-d_5_ and d18:0 Sph-d_5_ were quantified *via* d18:0 Sph, whereas calibration curves of Cer d18:0/16:0 and Cer d18:1/16:0 were used to quantify Cer d18:0-d_5_/16:0-d_3_ (C16 dhCer-d_8_) and Cer d18:1-d_5_/16:0-d_3_ (C16 Cer-d_8_), respectively. Cer d18:1/17:0 and d17:0 Sph were used as internal standards. Bars represent mean concentrations (detailed in the main text) ± SEM (^∗∗∗^*p* < 0.001; n.s., non-significant; *n* = 5). Additionally, individual values of the replicates are depicted as circles.

Intra-assay variability was assessed in a separate experiment using a new batch of rat liver microsomes. “Standard conditions” plus NADH were applied to five technical replicates. In addition to NADH, FB_1_ was added to another five replicates. *De novo* formed deuterated sphingolipids were quantified by external calibration using unlabeled analogs as reference compounds (concentrated from 0 to 1000 nM), as well as d17:0 Sph and Cer d18:1/17:0 as internal standards. As can be seen from Figure 8, the intra-assay variability (expressed as average % CV) was low, accounting for 11% and 15% for samples with or without FB_1_ addition, respectively. In the final sample volume (100 μL) the mean concentrations of 3KS-d_5_, d18:0 Sph-d_5_, C16 dhCer-d_8_, and C16 Cer-d_8_ were, respectively, 39.3, 201.3, 21.6, and 0.94 nM (“standard conditions” plus NADH). Addition of FB_1_ did not affect the formation of 3KS-d_5_ (30.4 nM) but did significantly increase d18:0 Sph-d_5_ (447.7 nM). Consistently, C16 dhCer-d_8_ and C16 Cer-d_8_ were reduced to 28% (6.1 nM) and 18% (0.17 nM). Taken together, using aliquots of identical batches of microsomes and reagents, the presented assay is reproducible. Larger variations are expected when different microsomal preparations are used due to deviating enzyme activities. However, this is precisely the intended field of application for the here established assay: the study of altered sphingolipid *de novo* synthesis in microsomal fractions of defined origin.

## Discussion

Cer are sphingolipid species central to sphingolipid metabolism and play key roles in the pathogenesis of a variety of human diseases. Modification to Cer metabolism has been linked, among others, to the onset of various cancers, type 2 diabetes, Alzheimer’s disease, and major depression (Kurz et al., 2019). An important metabolic pathway through which Cer are formed in the cell is *de novo* synthesis within the ER. It is, therefore, consistent that therapies targeting the modulation of enzymes involved in *de novo* synthesis have been developed for diseases associated with Cer dysregulation. For instance, it has been shown that myriocin, a potent SPT inhibitor, suppresses murine melanoma growth (Lee et al., 2012) and mediates the regression of atherosclerotic lesions in hyperlipidemic apolipoprotein E knockout mice (Park et al., 2008). Efforts have also been made to develop specific CerS inhibitors as novel therapeutic treatment options (Schiffmann et al., 2012), one of which was recently used to elucidate the role of CerS1 and its product Cer d18:1/18:0 (C18 Cer) in the storage of dietary lipids (Turner et al., 2018). Accordingly, methods have been developed to determine the activities of individual enzymes involved in the biosynthesis of sphingolipids, and various activity assays for SPT (Rütti et al., 2009), KDSR (Fornarotto et al., 2006), CerS (Couttas et al., 2015), and DEGS (Munoz-Olaya et al., 2008) are now available. Up to this point in time, however, there existed no cell-free assay capable of concurrently assessing the activities of all four enzyme families involved in sphingolipid *de novo* synthesis. A prerequisite for such an assay would be the specific tracking of SPT substrates as they are incorporated into the Cer framework. One approach for this would be the use of exogenous enzyme substrates alien to eukaryotic cells (e.g., carrying odd-numbered carbon chains or stable-isotope labels) and highly specific and sensitive detection by means of mass spectrometry.

Ren and co-workers developed an *in vitro* SPT activity test system utilizing yeast microsomes treated with palmitoyl-CoA and deuterated L-serine as SPT substrates. By means of HPLC-TQ MS, they were able to follow the incorporation of the labeled amino acid into 3KS and d18:0 Sph, which they used to determine SPT kinetics in yeast (Ren et al., 2018). More recently, Harrison et al. used differentially labeled L-serine isotopologs and either palmitoyl-CoA or pentadecanoyl-CoA as SPT substrates to study the impact of stable-isotope label number and position in L-serine on the kinetics of recombinant human and bacterial SPT. For this, they applied HPLC equipped with a fluorescence detector to quantify C17 LCBs, and direct infusion high-resolution MS for detection of 3KS products (Harrison et al., 2019). Both studies used state-of-the-art techniques but limited their scopes to the first two steps of sphingolipid *de novo* synthesis. Another recently published study presented a more complex approach that allows comprehensive monitoring of sphingolipid metabolism, not restricted to the ER. The authors tracked the flux of d17:0 Sph through the sphingolipid metabolism of MCF-7 human breast adenocarcinoma cells in an one-step *in situ* assay using HPLC-TQ MS. Data on complex sphingolipids carrying a C17 sphingoid base backbone, such as dhCer, Cer, sphingomyelin, and hexosylceramide, were presented (Snider et al., 2018). However, the choice of d17:0 Sph as a starting material inevitably excludes the first two steps of *de novo* synthesis, catalyzed by SPT and KDSR. Strategies for monitoring sphingolipid metabolism in mammalian cells have recently been reviewed (Snider et al., 2019). To the best of our knowledge, the here presented method is the first reported cell-free assay capable of assessing *de novo* sphingolipid synthesis in its entirety. This results in two major innovations applicable to this field of research. First, the complete sphingolipid *de novo* synthesis can now be monitored in tissues or cells of choice, *ex vivo*. Second, our assay is the first that allows investigation of the complete ER-based metabolism of atypical or chemically tailored sphingoid bases, molecules that are of growing interest to the field.

We have established a protocol that uses rat liver microsomes as a source of enzymes, and palmitate-d_3_ and serine-d_3_ as initial labeled substrates for sphingolipid synthesis. Thus, unlike other published SPT activity assays, we use a non-activated fatty acid as our substrate precursor. This strategy requires *in situ* fatty acid activation, for which we added CoA and ATP to the microsomal fraction containing acyl-CoA synthetases (Normann et al., 1981). This procedure offers several advantages. First, we are able to test atypical SPT substrates – e.g., other fatty acids or derivatives – and so are not dependent on commercially available acyl-CoAs. Decades ago, it was described that the (rat liver) SPT affinity to fatty acyl-CoAs is rather broad and not restricted to the endogenous substrate palmitoyl-CoA (Williams et al., 1984). Likewise, it would be possible to change the amino acid precursor. For instance, if L-serine-d_3_ were replaced by L-alanine or L-glycine (preferably labeled) while palmitate-d_3_ is maintained, the formation of deuterated 1-deoxysphingolipids or 1-deoxymethylsphingolipids can be monitored, respectively (Duan and Merrill, 2015). Another advantage of this procedure is that all molecular requirements to monitor the *N*-acylation of d18:0 Sph catalyzed by CerS are met simultaneously. In this third step of sphingolipid biosynthesis, acyl-CoAs of different chain lengths serve as substrates for six different CerS isoforms (Figure 1). The addition of ATP and CoA to the assay also would permit the observation of different fatty acids as they were incorporated into the developing Cer backbone. In fact, the *de novo* formation of C16 dhCer-d_8_ from the assay substrates palmitate-d_3_ and serine-d_3_ (Figure 5) confirmed the activity of CerS5 and CerS6 in our assay, since these isoforms prefer palmitate (Levy and Futerman, 2010) and, thus, palmitate-d_3_ as a substrate. It would be straightforward to add the preferred fatty acids of other CerS isoforms, with or without labels, to increase the complexity of the assay and to assure even more detailed insights into sphingolipid *de novo* synthesis. In the present study, however, we focused on labeled physiological SPT substrates. In the future, follow-up studies with atypical SPT substrates, a broader spectrum of CerS substrates (all preferably stable-isotopically labeled), and combinations of these are envisaged. From an analytical point of view, the labeling of both the fatty acid and amino acid precursor molecules with stable-isotopes is advantageous. Most published MS-based SPT assays used labeled serine only (Ren et al., 2018; Harrison et al., 2019). Since serine is a small molecule (chemical formula: C_3_H_7_NO_3_), the number of introducible labels is limited. Taking into account the losses of α-deuterium and ^13^C-isotope at the carboxylic acid group (depending on the labeled serine reference substance used) during SPT catalysis (Figure 3), the labeling may be too low to avoid interferences with unlabeled assay components. More precisely, the use of labeled serine in combination with unlabeled palmitoyl-CoA, as done in the Ren et al. (2018) study using L-serine (2,3,3-d_3_), leads to the formation of 3KS-d_2_ as the initial SPT product. Following the *de novo* synthesis under these conditions, Cer d18:1-d_2_/16:0 (C16 Cer-d_2_) would be formed as the consistent product. Using a TQ MS for detection (as done in the above-mentioned study) will inevitably cause interferences with unlabeled Cer d18:1/16:0 (C16 Cer) present in the microsome preparation, due to the low mass accuracy of the quadrupole mass analyzer. The natural isotope pattern of the molecular ion [M-H_2_O+H]^+^ of C16 Cer is as follows (relative abundances in parentheses): (*m/z*) 520.5088 (100%), 521.5122 (36.8%), and 522.5155 (3.9%). The monoisotopic (exact) mass of C16 Cer-d_2_ is *m/z* 522.5214. With a TQ MS it is not possible to resolve the mass difference between the two nominally equal masses (*m/z* 522.5155 for C16 Cer and *m/z* 522.5214 for C16 Cer-d_2_). Since C16 Cer is present in microsomes in significant amounts, as confirmed by our QTOF raw data (not shown), misidentification of labeled, *de novo* formed Cer is very likely. For these reasons, we performed our assay with both labeled fatty acid and labeled amino acid to increase the deuteration level of the metabolites (products identified in the current study were five to eight-times labeled; see Figures 2, 4–6). Furthermore, besides TQ MS as a very sensitive detection system, we also used QTOF MS, a technique with high mass accuracy. Thus, we can unequivocally distinguish between *de novo* formed products and intrinsic sphingolipids present in the microsome preparation. Recently, Harrison and co-workers found that the presence of a serine α-deuterium significantly decreased the reaction rate of recombinant bacterial SPT, a point also applicable to L-serine (2,3,3-d_3_) used in the present study. However, recombinant human SPT was unaffected by the deuterium label at Cα position (Harrison et al., 2019). We used rat liver-derived SPT, which has a greater homology to human SPT than that of bacterial SPT. Nonetheless, testing of differently labeled serine substrates seems worthwhile and is planned for the future.

With our established sphingolipid *de novo* synthesis assay, we have identified four distinct and sequential metabolites, namely 3KS-d_5_, d18:0 Sph-d_5_, C16 dhCer-d_8_, and C16 Cer-d_8_. The latter two have, as yet, not been demonstrated using comparable test strategies. The use of enzyme inhibitors and varied cofactor quantities validated our approach. The following observations (illustrated in Figure 7) are in agreement with the well-studied progress of the sphingolipid biosynthesis in the ER (Gault et al., 2010): (1) *de novo* synthesis was abolished in the presence of the SPT inhibitor myriocin, (2) omission of reducing agents (NADPH/NADH) caused *de novo* synthesis to halt after SPT reaction (in conjunction with highest 3KS-d_5_ signal intensity), and (3) addition of the CerS inhibitor FB_1_ drastically reduced the *N*-acylation of d18:0 Sph-d_5_ (in agreement with its highest signal response) and thus the abundance of labeled dhCer and Cer. Two further observations need to be addressed. Firstly, assay supplementation with NADPH and NADH increased detected amounts of d18:0 Sph-d_5_, C16 dhCer-d_8_, and C16 Cer-d_8_ compared to addition of NADPH alone (in line with a 3KS-d_5_ signal reduction). This effect was particularly pronounced for dhCer and Cer, which were almost doubled. Microsomal preparations used for metabolism studies are usually supplemented with reducing agents. Under “standard conditions,” we added a threefold excess of reducing equivalents (1 mM NADPH) compared to the conversion limiting substrate palmitate-d_3_ (0.3 mM). This excess was doubled when a surplus of 1 mM NADH was added. It is therefore unlikely that under “standard conditions,” the amount of reducing agent was insufficient for quantitative conversions. At least for the DEGS-catalyzed step of sphingolipid biosynthesis in rat liver microsomes, it has been reported that NADPH and NADH function equally well as cofactors (Michel et al., 1997). Since the addition of NADH appears to have a positive effect on the first reductive step, the formation of d18:0 Sph-d_5_, our results give rise to more detailed investigations. The second aspect of our data to be discussed, and a possible limitation of this assay, is the relatively small amount of C16 Cer-d_8_ detected. As outlined before, we are the first to track the incorporation of palmitate and serine into the Cer core-structure of complex sphingolipids in a structure-specific manner within a cell-free system. However, the obtained ratio between detected Cer and its direct precursor dhCer is in contrast to published lipidomics data (Barbarroja et al., 2015; Mielke et al., 2015; Hernández-Tiedra et al., 2016). For instance, in healthy humans, molar Cer/dhCer ratios in both serum and liver account for approximately 10:1 (Apostolopoulou et al., 2018). Although lipidomics data are subject to a certain degree of variability, depending on species, tissue, body fluid, cell line etc. studied, the Cer/dhCer ratio found in our assay was 0.044 (Figure 8, white bars) and is therefore substantially lower than previously reported. Reasons for this could be extraordinarily high CerS activity and/or low DEGS activity. Since *in vitro* systems are rarely more metabolically competent than living organisms, the former seems unlikely. Loss of DEGS activity in the microsomes is probably the most plausible explanation, although the reasons for this remain speculative. There may have been some mechanical phenomena in the production of microsomes that interfere with DEGS activity, but not those of SPT, KDSR and CerS. However, all four enzyme families are localized adjacent at the ER membrane with their catalytic sites facing cytosolically (Yamaji and Hanada, 2015), making the impairment of DEGS alone implausible. An inadequate provision of cofactors can also be excluded for reasons described above. Various rat liver microsome-based DEGS activity assays have been published (Michel et al., 1997; Munoz-Olaya et al., 2008; Rahmaniyan et al., 2011). Unlike these, our assay applies the substrates of an enzyme located three steps upstream in the *de novo* synthesis pathway, rather than those directly relevant to DEGS activity. Thus, inhibitory effects of upstream sphingolipid metabolites or assay components cannot be ruled out. Possibly, the lack of Cer trafficking into other cell compartments (e.g., Golgi apparatus) contributes to the lower amounts of labeled Cer d18:1/16:0 formed in the presented microsomal approach. To account for the possibility that ER-resident alkaline ceramidases (ACERs) (Coant et al., 2017) hydrolyzed the *de novo* formed Cer d18:1-d_5_/16:0-d_3_, we analyzed the lipid extracts for d18:1 Sph-d_5_ (*m/z* 305.3 → 287.3). In addition, we searched for Cer t18:0-d_5_/16:0-d_3_, as the DEGS2 isoform, typically found at low levels, has been shown to catalyze C4-hydroxylation (without Δ4-desaturation) of the d18:0 backbone (Mizutani et al., 2004; Omae et al., 2004). According to Sullards et al. (2011), ceramide species containing a 4-hydroxysphinganine (t18:0) base also produce the *m/z* 264.3 fragment specific for ceramides. Hence, we analyzed our samples for the adjusted mass transition *m/z* 564.6 → 269.3 (deuterium labels on t18:0 backbone and fatty acid moeity). However, we were unable to detect either d18:1 Sph-d_5_ or Cer t18:0-d_5_/16:0-d_3_ in our assay. Further studies should be conducted to shed light on this issue.

## Conclusion

In conclusion, we have developed a microsomal *in vitro* assay to study the entire *de novo* synthesis of stable-isotopically labeled sphingolipids by HPLC-MS(/MS). Obtained results are in agreement with the proposed cascade of sphingolipid biosynthesis taking place in the ER. The presented methodology with low intra-assay variability may serve as a useful tool for monitoring alterations in sphingolipid *de novo* synthesis in the microsomal preparations of cells or tissues. Doing so, it is possible to track natural SPT substrates or downstream metabolites, as well as sphingolipid analogs that are of increasing importance to the field. For instance, the visualization of biomolecular processes, physiological or pathophysiological in nature, in which sphingolipids are involved, is an especially important area of discussion in current literature. In this regard, a useful tool is “click chemistry,” in which functionalized, tailor-made sphingolipid derivatives can be coupled with fluorophores (or other labels) and thus made visible (Fink and Seibel, 2018). The here presented assay in combination with mass-specific detection will be of substantial importance for such studies, as it is now possible to investigate whether customized sphingolipids are metabolized in the ER and, if so, at which specific step. This will be crucial in estimating their cellular fates and physiological impacts. Likewise, our method may be applied to a variety of different natural and hypothesized LCB analogs that have been synthesized in order to study the metabolism of disease-related 1-deoxysphingolipids (Saied et al., 2018). Furthermore, the efficacy of newly designed inhibitors of enzymes involved in *de novo* sphingolipid synthesis can be tested with our protocol.

## Data Availability Statement

All datasets generated for this study are included in the manuscript/supplementary files.

## Ethics Statement

The animal study was reviewed and approved by the Landesamt für Arbeitsschutz, Verbraucherschutz und Gesundheit Brandenburg.

## Author Contributions

DW, EG, BK, and FS conceptualized the study and interpreted the data. DW performed the *in vitro* experiments. FS was responsible for LC-MS analytics and wrote the manuscript with the help of DW and BK. All authors have read and approved the manuscript.

## Conflict of Interest

The authors declare that the research was conducted in the absence of any commercial or financial relationships that could be construed as a potential conflict of interest.
